# 2‐Octyl cyanoacrylate adjuvant use in calcaneal tendon repair: A comparative experimental study in rabbits

**DOI:** 10.1002/jeo2.70222

**Published:** 2025-04-01

**Authors:** Rogério de Andrade Gomes, Marco Antônio Percope de Andrade, Luiz Eduardo Moreira Teixeira, Bruno Jannotti Pádua, Sidney Max E. Silva, Matheus Maciel Vilela, Paulo Feliciano Sarquis Dias, Eduardo Paulino Júnior, Ivana Duval de Araújo

**Affiliations:** ^1^ Department of Orthopedics and Traumatology Hospital Francisco José Neves (Unimed‐Belo Horizonte) Belo Horizonte Minas Gerais Brazil; ^2^ Department of Locomotor Apparatus UFMG Belo Horizonte Minas Gerais Brazil; ^3^ Department of Anatomic Pathology UFMG Belo Horizonte Minas Gerais Brazil; ^4^ Department of Surgery UFMG Belo Horizonte Minas Gerais Brazil

**Keywords:** calcaneal tendon, cyanoacrylate, rupture, sutures, tendon injury, tendon repair

## Abstract

**Purpose:**

The treatment of Achilles tendon (AT) rupture can be conservative or surgical; the latter is better indicated in high‐performance athletes. The pursuit of faster recovery and reductions in skin complications and re‐ruptures have improved the tendon repair technique and early assisted rehabilitation programs. Our goal was to check whether or not the adjuvant use of 2‐octyl cyanoacrylate (CA) provides additional mechanical resistance to the calcaneal tenorrhaphy when compared to sutures alone.

**Methods:**

In the study, 45 rabbits were used, and distributed into three groups of 15 animals each. They underwent two surgical procedures at a 30‐day interval. In the first, a complete AT section was done on the right side and then was repaired using three different techniques: suture alone in Group 1, suture with 2‐octyl CA in Group 2 and 2‐octyl CA alone in Group 3. After 30 days, the rabbits were euthanized, and their right‐side calcaneal tendon was resected, and sent the specimen for pathology analysis. Then, a cross‐section of the left calcaneal tendon was performed, and the same three repair techniques were used. After 15 min, the repaired tendons to the linear traction mechanical strength test were done.

**Results:**

The results from the linear strength test showed that suture with glue outperformed suture alone (*p* = 0.0427), and glue alone (*p* < 0.0001). The pathology results were evaluated according to inflammation, repair process and tendon thickness. The report mentioned a high‐intensity mixed inflammatory pattern (acute and chronic), outstanding neovascularization, tissue necrosis and abscess in most of the cases that used CA. However, there were no local or systemic clinical signs of an infectious process.

**Conclusions:**

2‐octyl CA added immediate mechanical strength to the calcaneal tendon repair in the rabbits, without increasing local complications, despite pathological changes up to 30 days after the repair.

**Level of Evidence:**

NA study.

**Abbreviations**
AT: Achilles tendon
CA: cyanoacrylates
H&E: haematoxylin–eosin
N: Newton
UFMG: Federal University of Minas Gerais

## INTRODUCTION

The Achilles tendon (AT) is the strongest tendon in the human body [2, 22]. In some exercises, it may be able to support a load greater than 12 times its body weight [14]. However, it is more likely to suffer injuries related to these activities, such as inflammatory, degenerative or mixed tendinopathy, which may culminate with its rupture. The incidence of this injury has increased in recent decades, especially with the intensification of sports practice, accounting for 44%–83% of ruptures [21].

The aetiology of these diseases is multifactorial, encompassing factors related to chronic overload on a deficient support structure, vascular deficiency (in the middle of the tendon), patients' intrinsic factors (hindfoot alignment, genetic factors), use of medications such as corticosteroids and fluoroquinolones, and also to failure in the healing process of microlesions [7, 15, 18, 23, 33].

AT rupture is the utmost expression of tendon diseases, resulting from a chronic degeneration process that is often asymptomatic. It occurs at an average incidence of 8.3 per 100,000 people, peaking between the fourth and fifth decades of life [34].

High‐demand patients, not only athletes but also those with above‐average recreational sports practice, are better managed by surgery, necessitating shorter immobilization periods and earlier rehabilitation onset, leading to an earlier return to work.

Cyanoacrylates (CAs) were first described in 1949. Surgical glues emerged as an alternative to suturing and have been in medicinal use for over 30 years [8]. They have been employed in various medical procedures, including skin lesions, nail bed repairs, corneal lesions, treatment of varicose veins, maxillofacial procedures, gynaecological procedures (tubal occlusion) and neurosurgery for intracranial aneurysms [1, 10, 11, 19, 24, 35]. One type of glue, isobutyl CA, was compared to the conventional suture and to the suture with glue in the calcaneal tendon of rabbits, showing that the suture associated with the monomer was better after a stress rupture test [6]. A further study indicated that the combination of N‐butyl‐2‐cyanoacrylate with suture is superior to epitendinous reinforcement with 3‐0 prolene [27].

A long‐chain monomer developed to overcome deficiencies in its short‐chain derivatives, 2‐octyl CA, polymerizes with an exothermic reaction upon contact with a basic medium or fluid. With a breaking strength up to four times that of its precursor, N‐butyl‐2‐cyanoacrylate, it exhibits slow decay, low inflammatory reaction, and less histotoxicity, forming a smaller amount of formaldehyde [13]. Its breaking strength is comparable to that of a 4‐0 nylon suture [9]. The bond becomes robust in 45–60 s, reaching full strength in 2 min and 30 s. US Food and Drug Administration has been approved since 2001 for repairing small skin lesions, and 2‐octyl CA offers advantages in terms of strength and reaction profile.

Various adjuvant substances have been tested to enhance the mechanical resistance of the system, including fibrin glue (BioGlue™) and several CAs [17, 38]. However, most were unsuccessful in increasing resistance. The medical literature lacks studies demonstrating the effectiveness of biomaterial adjuvants in tenorrhaphies.

The purpose of this study is to verify whether the adjuvant use of 2‐octyl CA offers additional resistance to calcaneal tenorrhaphy compared to isolated suturing using the same technique.

## METHODS

This study involved 45 male New Zealand adult rabbits, approximately 3 months old, obtained from the Farm of the Veterinary School of the Federal University of Minas Gerais (UFMG). Housed individually in cages with ad libitum access to water and food, the rabbits were kept in a well‐ventilated area with controlled temperature, ventilation and a natural day–night cycle. Clinical signs and animal welfare were evaluated by their behaviour, normal acceptance of food and reaction to contact with the team. The cages were 50/40/53 cm in size (height/width/depth) and were located in a single room in the vivarium, housing that species only.

This study was proposed and developed following the conditional guidelines by Arrive 2.0, in order to provide complete and transparent information about the way in which it was conducted [28]. All items in the guidelines, such as study design, sample size determination, outcome measurements, statistical methods and experimental procedures were followed and described as required for conducting studies in animals.

The study design consisted of a control group and two other groups for comparison. The sample size was based on analysis with statistical power of 80% and following the principles of the 3Rs (replacement, reduction and refinement) [29].

To minimize selection bias, animals in the three groups were matched for age and weight, all being male. All procedures, including surgeries and daily care, were conducted by the same professional. Euthanasia was performed with the least possible suffering for the animals.

The sample size was calculated using the analysis of variance method for independent groups, with alpha = 0.05 and statistical power of 0.80. After performing a sample calculation with a power of 0.80, a number of 13 rabbits per group were reached and were used 15 animals per group, for a total of 45 animals, considering the probable loss in some group due to illness or some other reason.

Our study was approved by the Research Ethics Committee under the Protocol of Ethics in the Use of Animals, protocol number 390/2017 from 21 May 2018. The study started in August 2018, with the operational part (bench‐work) on 20 September 2018 with the first group.

### Study design

The animals were randomly allocated into three groups:

Group 1: (*n* = 15) suture only;

Group 2: (*n* = 15) suture of the lesion associated with glue; and

Group 3: (*n* = 15) glue alone.

The first part of the surgery was performed on the right side of all the rabbits, with a 1‐week interval between the groups.

The animals were anaesthetised and kept under sedation by intramuscular injection of xylazine (Xilazin®; Syntec) at a dose of 5–10 mg/kg and ketamine (Cetamin®, Syntec) at a dose of 40 mg/kg. During induction (10 min before skin incision), 50 mg/kg of cefazolin (Kefazol®: Antibiotics do Brasil) was also intramuscularly administered for wound infection prophylaxis. After the anaesthetic procedure, the rabbits were placed in the prone position and subjected to asepsis of the limb to be operated, with a degerming and alcoholic chlorhexidine solution.

The surgical incision was made in the right lower limb via a longitudinal, lateral paratendinous approach of approximately 4.0 cm. After dissecting and exposing the calcaneal tendon, it was cross‐sectioned (2.5 cm from its insertion) with a number 15 scalpel.

**Figure 1:**
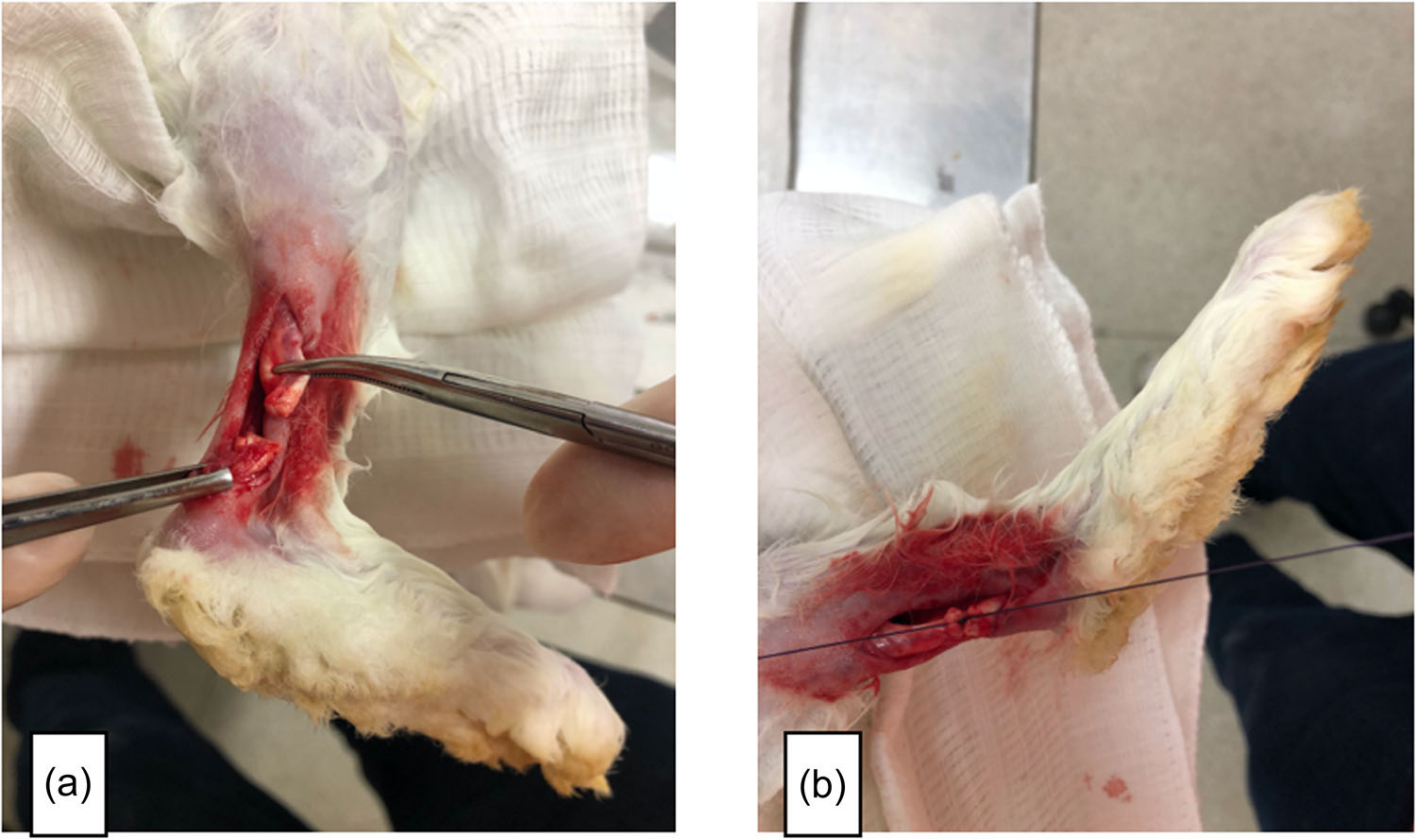
(a and b) Approximating the tendinous stumps and suturing them.

**Figure 2:**
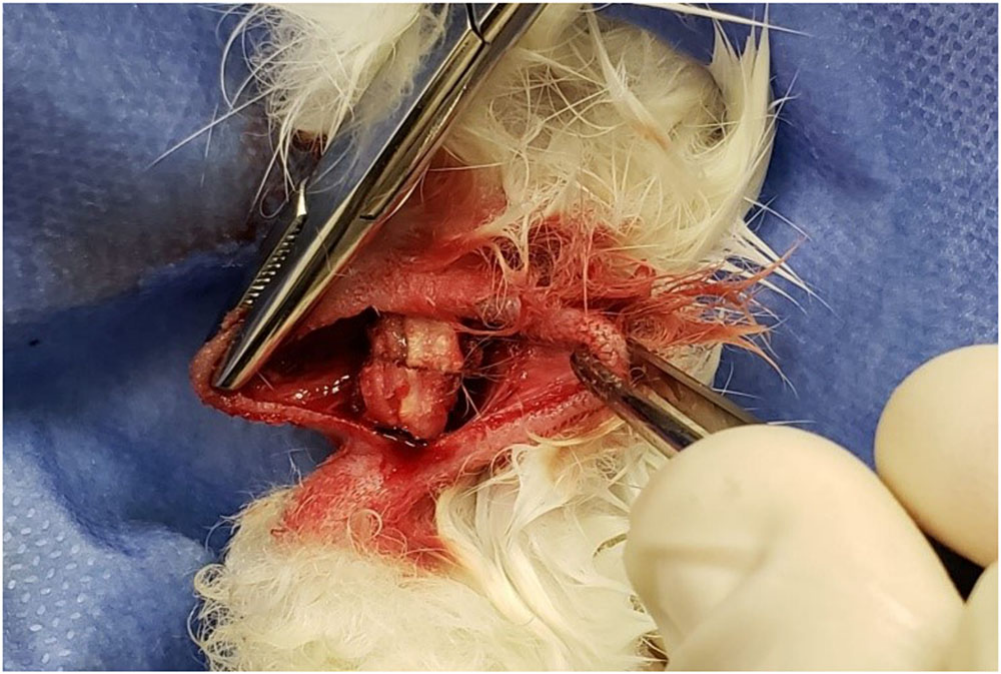
Final aspect of a tendon from the glue‐alone group.

After tenotomy, the stumps were approximated and submitted to tenorrhaphy using the modified Kessler suture, with polyglactin 910 absorbable suture (Qualtrus® 2‐0; Ethicon, Johnson & Johnson do Brasil) in Groups 1 and 2 (Figure 1a,b). Each tenorrhaphy received a suture thread, complemented with two more epitendinous stitches with the same suture thread. In Group 2 after suturing, and in Group 3 after approximating the stumps (Figure 2), 0.25 mL of 2‐octyl CA (Dermabond®; Ethicon, Johnson & Jonhson) was applied around the entire circumference of the tendon with a syringe, isolating it from the rest of the soft tissue, taking care not to let the glue come into contact with the skin and with the deep soft tissue. After bringing the stumps closer with the three different techniques, aforementioned, the skin was sutured with a 2‐0 nylon suture (Nylpoint®; Point Suture).

### Study sequence

Rabbits were operated on at 1‐week intervals and euthanized after 30 days. Rabbit number 06 from Group 1 developed prostration, irritability, an aggressive reaction to stimuli, and an injury in the gluteal region, interpreted as an infection. The rabbit was treated with antibiotics, showing subsequent clinical improvement. To maintain the integrity of the study, this animal was excluded.

Initially, the rabbits were weighed, and the data were recorded. The euthanasia was done in a 40% carbon dioxide (CO_2_) chamber, after sedation with 15 mg midazolam, having two rabbits placed in the chamber at a time. After 4–6 min, the animals progressed to cardiorespiratory arrest and death.

After death, the animals were submitted to a wide incision in the posterior region of the operated leg to remove the musculotendinous unit of the sural triceps from the proximal part of the muscle to the end part of the calcaneal tendon.

The initial evaluation consisted of a visual inspection, looking for adhesions between the tendon, the skin and deep structures. Then, the material from the suture stumps was sampled and sent for a pathology study. This process was done with a 3.0 cm segment that encompassed the immediate proximal and distal parts of the tendon and the tenorrhaphy site, resected with the aim of assessing tissue reaction to suture and glue, as well as interferences in the healing process.

The resected pieces were placed in properly identified individual vials, soaked in a 10% buffered formalin solution and then sent for analysis at the Pathology Anatomy laboratory, set in paraffin blocks and cut longitudinally with a microtome, and dyed with haematoxylin–eosin (H&E). The analysis of the routine H&E examination was considered sufficient by the pathologist. The use of other histological techniques would increase the cost of the project and would not add information different from that already evaluated in H&E. The central part of the repaired area tendon was studied. The resected pieces were assessed using pathological parameters of inflammation and repair. The semiquantitative analysis of inflammation took into account the type of inflammation (if absent, acute, chronic or mixed), the intensity of the process (absent, sparse, mild, moderate or severe), its distribution (no inflammation, focal, multifocal or diffuse) and having an abscess or not. A pathologist with over 20 years of experience led the study and was responsible for scoring the macroscopic appearances of the tendons and histopathology. He was unaware of the groups from which the samples came.

The semiquantitative analysis of the repair assessed: fibrosis (absence, mild fibrosis, moderate or severe); neovascularization (absent, mild and focal, mild and diffuse, moderate and severe); granulomatous formation (absent, present mild, moderate or severe); recent scar (absent or present) and cyst formation (absent or present). In addition, the measurement in millimetres of the resected segment and the inflammation and repair data were recorded for further study.

Initially, a pathology study of the normal AT of two rabbits was done to define the normal histology for this structure, evaluating the shape and disposition of the fibrils and fibre bundles, their cellularity and vascular pattern. The previous study of normal tendons of two rabbits served as a reference to compare with the operated tendons, making this semiquantitative study based on the changes described above. In general, the definitions were established as follows:

1. Mild changes—up to 50% above normal;
2. Moderate changes—between 50% and 100% above normal; and
3. Marked changes—two times above normal.

After this phase, the same procedures were performed on the contralateral limb (left side), studying the 15 animals in the three groups—suture alone, suture with glue and glue alone, with an interval of 1 week between the groups. The break strength was tested after the application of the three techniques in the contralateral limb. In the two groups to which CA was applied, the resected tendon was submitted to a resistance test in a linear tensiometer (manufactured by the Computer Science Department at the UFMG, Belo Horizonte, Brazil) 15 min after the final repair (for glue drying). For the resistance test, the myotendinous structure was removed, with the entire proximal myotendinous part in the distal segment, including a 1.0 cm fragment of the calcaneal bone. In the proximal segment, a Krackow‐type suture was done using a 5 polyester suture thread (Ethibond, Ethicon– Johnson & Johnson do Brazil), which was attached to the tension steel cable of the linear tensiometer. The insertional segment with the bone fragment was attached to the fixed claw at the base of the device.

The tensiometer recorded the rupture values in kilograms‐force, which were recorded for future data analysis and compared between the groups. The values were later converted to Newtons (N), a unit of force commonly used in reference publications.

The rabbit carcasses were separated and collected for burial in wooden boxes on appropriate burial grounds.

### Statistical analysis

Initially, an exploratory analysis was conducted to determine data normality using the D'Agostino–Pearson and the Shapiro–Wilk tests. These tests calculated to what extent each value differed from the expected value within a Gaussian distribution (parametric distribution).

According to the nature of the data, the mean and standard deviation values were obtained as standard deviation (for parametric data, i.e., with normal distribution—weight) or median (25th and 75th percentile values, for the non‐parametric data—break strength). The results of all analyses were plotted in tables and graphs, and the differences obtained were considered statistically significant when the *p* < 0.05. For the statistical analyses, the GraphPad Prism® software, version 5.0 for Windows, was used. For the non‐parametric data (break strength), the Mann–Whitney test was used to detect possible significant differences between the study groups (Group 1 × Group 2− Group 1 × Group 3). To look for possible significant differences, the data were submitted to the outliers detection test. The GraphPad QuickCalcs Outlier Calculator software (http://graphpad.com/quickcalcs/Grubbs1.cfm) was used. In Group 1 (suture alone), an outlier value (Sample 14—break strength) was found, which was excluded from the analysis. The parametric data (weight) were submitted to the Student's *t* test (Group 1 × Group 2 − Group 1 × Group 3).

## RESULTS

### Descriptive and comparative analysis (weight)

There was no statistical difference between the studied groups in terms of weight. The mean value found in Group 1 was 2929 g (±257.80); in Group 2, it was 2948 g (±324.70); and in Group 3, it was 2965 g (±174.50), showing homogeneity in the experimental groups.

### Pathology analyses

The resected parts were assessed using the pathological parameters of inflammation and repair. The measurement in millimetres of the resected segment was recorded with the inflammation and repair data. Table 1 depicts this information.

**Table 1:** Pathological alterations of the resected parts.

Group‐rabbit	Inflammation				Repair					Measure
	Type	Intensity	Distribution	Abscess	Fibrosis	Neovascularization	Granuloma	Recent scar	Cyst formation	
G1 ‐01	0	0	0	0	3	1	0	0	1	4.78
G1‐02	2	1	1	0	4	2	1	0	0	4.07
G1‐03	2	2	2	0	3	3	1	0	0	5.67
G1‐04	2	2	2	0	3	4	1	0	1	9.15
G1‐05	2	1	2	0	4	4	1	0	0	5.74
G1‐07	1	1	2	0	2	2	0	1	1	5.82
G1‐08	2	1	2	0	4	4	1	1	1	6.55
G1‐09	1	1	1	0	4	4	0	1	0	7.73
G1‐10	1	1	1	0	3	3	1	0	0	4.44
G1‐11	3	2	2	0	3	3	1	0	0	3.88
G1‐12	3	2	2	0	3	4	1	0	0	6.61
G1‐13	3	2	2	0	4	4	1	1	1	6.56
G1‐14	1	1	1	0	4	3	1	1	1	2.69
G1‐15	3	1	1	0	4	3	1	0	0	3.73
G2 ‐01	3	3	2	1	4	3	3	1	1	4.12
G2‐02	3	3	2	0	4	4	3	0	0	5.88
G2‐03	3	2	2	1	4	4	2	0	1	4.98
G2‐04	3	3	3	1	4	4	3	0	0	6.2
G2‐05	3	3	2	0	4	4	2	1	0	5.45
G2‐06	3	3	3	1	4	4	3	0	0	7.35
G2‐07	3	3	3	1	4	4	3	0	0	5.62
G2‐08	3	4	2	1	4	4	3	0	1	8.88
G2‐09	3	4	3	1	3	4	3	0	1	6.3
G2‐10	3^a^	4	3	1	4	4	3	0	1	8.4
G2‐11	3	3	3	1	4	4	2	0	0	5.83
G2‐12	2	2	1	1	4	4	1	0	0	4.46
G2‐13	3	3	2	1	4	4	1	0	0	5.27
G2‐14	3	4	3	1	4	4	3	0	0	5.71
G2‐15	3	3	2	1	4	3	2	0	0	5.96
G3 ‐01	3	1	1	0	4	3	1	0	0	4.88
G3‐02	3	3	3	1	4	4	2	0	0	7.23
G3‐03	3	2	2	1	3	4	1	0	0	5.59
G3‐04	3	4	3	1	4	4	1	0	1	6.21
G3‐05	3	4	3	1	4	4	2	0	0	6.23
G3‐06	3	3	3	1	4	4	3	0	0	8.45
G3‐07	3	2	2	0	3	3	0	0	1	4.72
G3‐08	3	4	3	1	4	4	2	0	0	7.5
G3‐09	3	4	3	1	4	4	2	0	1	6.1
G3‐10	3	3	2	1	4	4	1	0	1	5.47
G3‐11	3	1	3	0	4	4	0	0	1	7.84
G3‐12	3	4	3	1	4	4	2	0	1	5.75
G3‐13	3	4	3	1	4	4	2	0	1	6.53
G3‐14	3	3	2	1	4	4	2	0	1	7.03
G3‐15	3	2	2	1	3	3	1	0	1	7.17

*Note*: Different colors were used to distinguish the three groups of rabbits. Group 1 is dark blue, group 2 is green and group 3 is light blue. Inflammation caption: Type: 0—absence; 1—polymorphonuclear (acute); 2—mononuclear (chronic); 3—mixed; Intensity: 0—no inflammation; 1—sparse; 2—mild; 3—moderate; 4—severe; Distribution: 0—no inflammation; 1—focal; 2—multifocal; 3—diffuse. Abscess: 0—Absent; 1—Present. Repair caption: Fibrosis: 0—no fibrosis; 1—mild fibrosis; 2—mild fibrosis; 3—moderate fibrosis; 4—severe fibrosis; Neovascularization: 0—absent; 1—mild and focal; 2—mild and diffuse; 3—moderate; 4—marked. Granulomatous reaction: 0—absent; 1—present and mild; 2—present and moderate; 3—present and marked. Recent scar: 0—absent; 1—present; Cyst formation: 0—absent; 1—present. Measurement caption: measurement of the tendon cross‐section at the repair site (in millimetres).

^a^ Pronounced inflammation, with skin fistulization.

**Figure 3:**
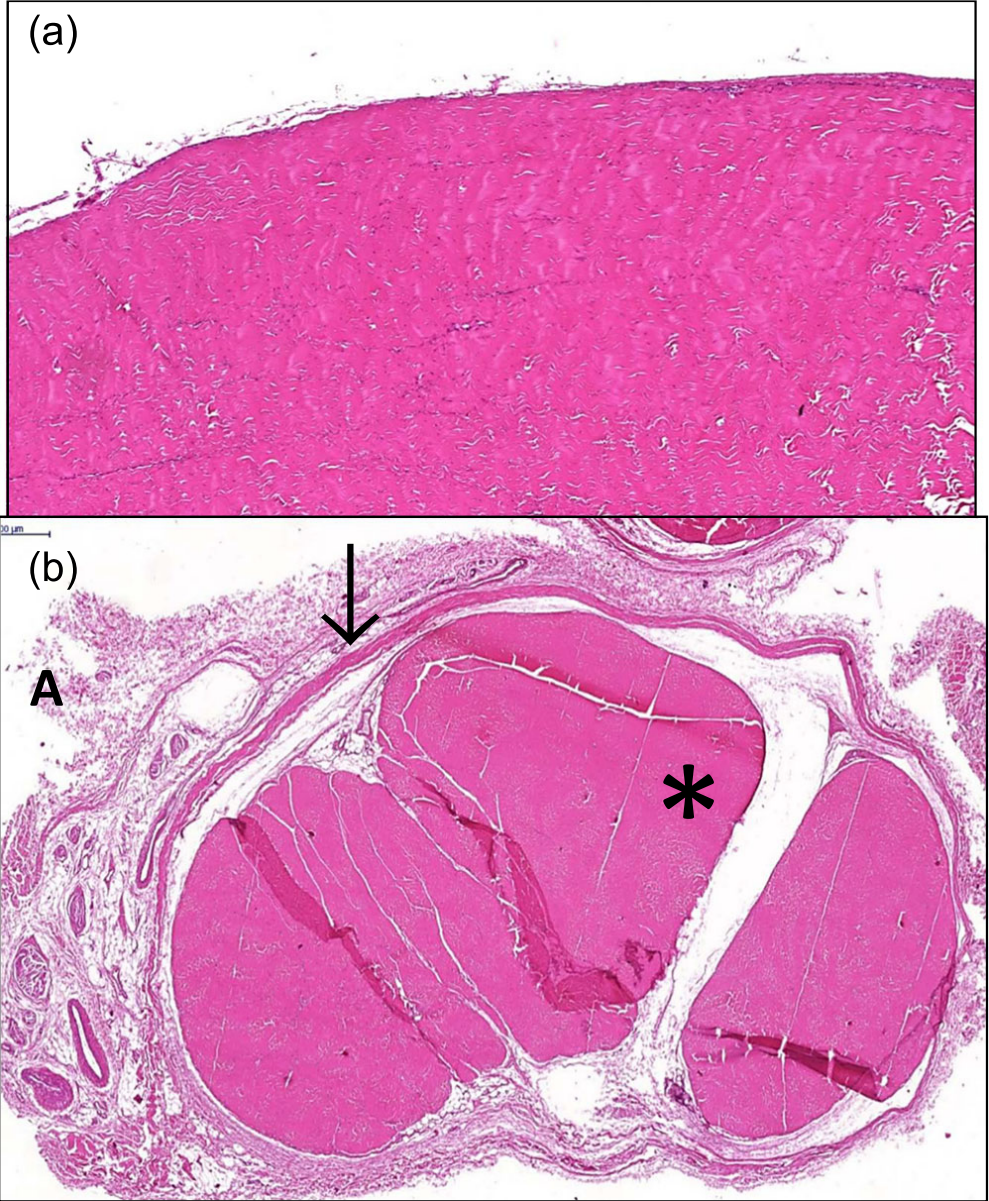
(a and b) Histological sections of normal calcaneal tendons. (a) Longitudinal section showing dense fibrous connective tissue, with fibres oriented in the same direction, eosinophilic. (b) Cross‐section showing the tendon sheath (arrow), with the epitenon (external) thicker and the endotenon (internal) looser, involving groups of tertiary muscle fascicles (*).

A pathology study of the normal calcaneal tendon of the two rabbits was done, as described previously. The study of normal tendons served as a reference to compare them with that of operated tendons, thus making a semiquantitative study based on the alterations found (Figure 3a,b).

**Figure 4:**
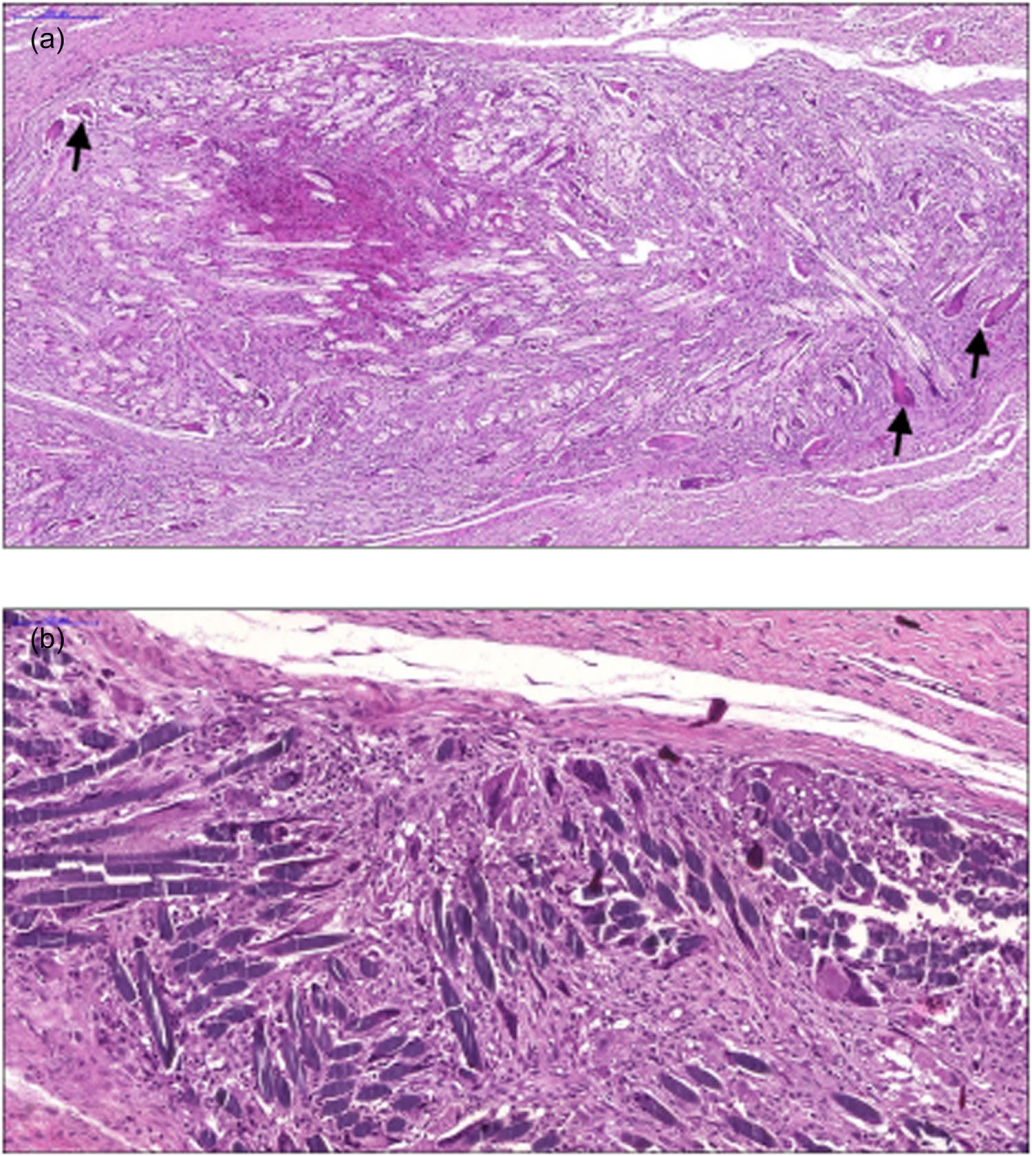
(a and b) Inflammation patterns: Group 1. (a) Giant cellular reaction to a foreign body, with granulomas containing histiocytes and multinucleated cells (arrows), involving suture residues. (b) Details, with connective tissue neoformation and inflammation involving the suture fibres, individually.

**Figure 5:**
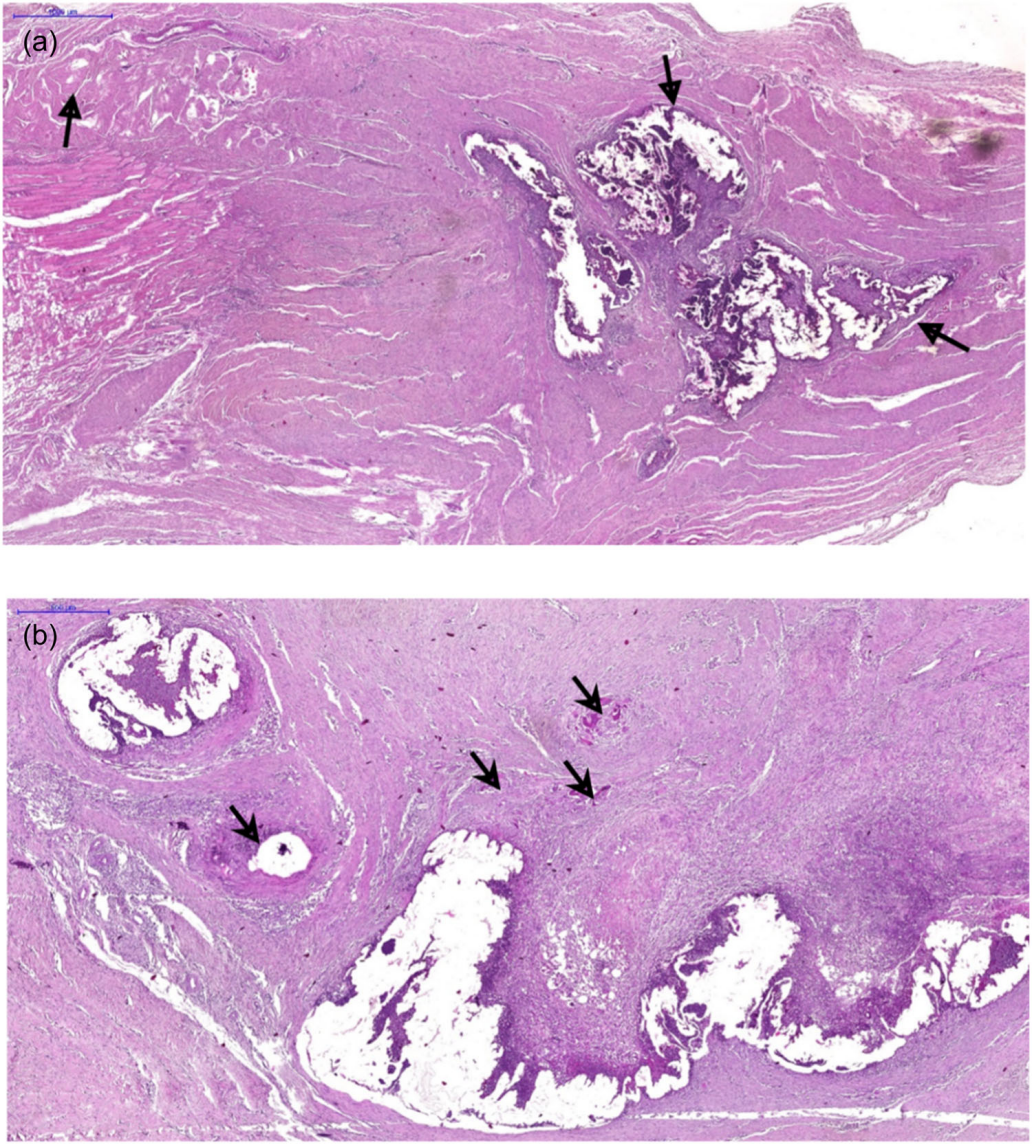
(a and b) Patterns of inflammation. (a) Areas of mixed inflammatory infiltrate, necrosis and formation of small abscesses. Note the connective tissue neoformation that surrounds the lesion (arrows). (b) Another area with the same pattern accompanied by a foreign body‐type granulomatous reaction (short arrows).

The inflammatory changes seen in the suture‐alone group were milder, without abscess formation and with non‐diffuse distribution of the process, when compared to the other two groups (Figure 4a,b). In Groups 2 and 3, which used CA, abscesses formed in 86.7% and 80% of the cases, respectively. A mixed inflammatory pattern (acute and chronic) was found in almost all the animals. Regarding the repair process, there was a more exuberant pattern of neovascularization in the groups in which the glue was used, as well as a granulomatous reaction of moderate/severe intensity (Figure 5a,b).

In the suture‐alone group, the granulomatous reaction was associated with reduced inflammatory activity and smaller granulomas, with a high degree of fibrosis. Fibrosis in this group was better organized, although there was still hypercellularity, with more straight fibres arranged in parallel, in the same orientation as normal fibres. Fibrosis in the cases in Group 2 was apparently more intense, with the tendons showing a mean diameter of 6.03 mm, close to those in Group 1 (5.53 mm). In Group 2, the areas of granulomatous reaction were larger, confluent and still with inflammatory activity, plus large areas of necrosis, often confluent, with the formation of small abscesses.

The fibrosis in Group 3 appeared more disorganized and even more intense when compared to Group 2. The mean diameter of the tendons was 6.45 mm (when normal it ranges between 3.23 and 5.49). Group 3 had some granulomatous reactions, of lower intensity than that seen in Group 2, but less frequent and with more intense inflammatory activity when compared to Group 1. With the number of animals used, there was no statistically significant difference in tendon thickness within the three groups.

The rupture results were compared using the linear traction test, in which the suture with CA group showed greater resistance than the control group (*p* = 0.0427) (Table 2); and the latter was better than the group in which glue alone was used for repair *(p* < 0.0001) (Table 3). Furthermore, there was no statistical difference in the calcaneal tendon break pattern between Groups 1 and 2 (Table 4). The monomer was able to add resistance to the system, perhaps acting directly between the tendon stumps and/or indirectly, increasing the adhesiveness at the thread/tendon interface. A comprehension of how the CA improved the system was not accomplished.

**Table 2:** Break strength analysis (Newton, N) in calcaneal tendons of rabbits submitted to different repair techniques.

Break strength (N)	Group 1	Group 2	*p*
	Suture alone	Suture + cyanoacrylate	
	*N* = 14	*N* = 15	
Median	51.62	70.86	0.0427
P25–P75	45.94–57.69	47.43–110.40	
Min–max	37.86–68.52	39.92–118.60	

**Table 3:** Break strength analysis (Newton, N) of calcaneal tendons of rabbits submitted to different repair techniques.

Break strength (N)	Group 1	Group 3	*p*
	Suture alone	Cyanoacrylate alone	
	*N* = 14	*N* = 15	
Median	51.62	12.84	<0.0001
P25–P75	45.94–57.69	9.64–21.98	
Min–max	37.86–68.52	7.80–35.85	

**Table 4:** Calcaneal tendon break pattern analysis of rabbits submitted to repair using suture alone (Group 1) and suture+ cyanoacrylate (Group 2).

Break pattern			
G1 (suture alone)	*f*	%	*p*
*Slippage*	14	100.00	1.0000^T^
Break	0	0.00	
Total	**14**	**100.00**	

G2 (suture + cyanoacrylate)	*f*	%	*p*
*Slippage*	10	66.67	0.0973^T^
Break	5	33.33	

## DISCUSSION

Tendinous sutures have two primary failure patterns: suture rupture (breakage or snapping) and suture slippage. The latter can lead to partial or total slipping of the stitch from the tendon structure, causing loosening or complete loss of the repaired structure. Various stitch types, such as locking or crossing sutures (e.g., Kessler and Krackow techniques), aim to reduce this risk. However, sutures with numerous crossings may induce ischaemia on the tendinous edges, compromising healing and forming a bulbar segment that can impede tendon sliding [40].

In addition to different surgical techniques, several studies aim to demonstrate the superiority of certain types of sutures or materials. The Krackow suture exhibits greater mechanical strength than the Kessler suture and is comparable to the Achillon® [20, 26, 30, 41]. The FiberWire® suture has superior mechanical resistance to Vicryl® (polyglactin) and Ethibond® (polyester), but its high cost makes it unaffordable for the Brazilian public healthcare system [16]. The polyester suture has higher strength but is associated with more tissue reaction. The number 1 polyglactin suture is widely used due to its low tissue reaction, cost‐effectiveness, good mechanical strength, and excellent retention capacity. Its break strength is maintained for 35 days, and it takes 8–10 weeks to be absorbed [5].

A study comparing the mechanical strength of knots with and without CA, reported superiority using CA, demonstrating that the type of rupture occurred differently in the groups [32]. It was only possible to equalize the break strength when multiple knots were used in the sutures of the group without glue. The sutures that used the glue failed due to thread breakage, while the sutures without the glue failed due to slippage. The results of the present study are in accordance with those from Bonutti et al., in which there was also an increase in the repair system resistance when adding CA [6]. However, in the latter study, the authors used a glue with lower break strength resistance (isobutyl CA) than the one used in the current study. In addition, they did not run a pathological evaluation of the operated tendons.

In studying tendon healing, several types of sutures, techniques and rehabilitation have been performed over the last decades, always seeking better results with a low rate of complications, early rehabilitation and a faster return to work and physical activities. In all these variables, it was possible to show strength superiority of some materials and better results with early assisted rehabilitation protocols and minimally invasive surgical procedures [12]. The decision to perform the repair with a modified Kessler suture, added by two complementary simple stitches using the 910 polyglactin thread, was based on the fact that the technique and material are also used in the repair of tendons of a similar diameter, such as flexor tendons, and human toe extensors.

The study sought to evaluate whether synthetic glue can increase the mechanical strength of the repair, either by providing more adhesiveness at the wire/tendon interface, or by creating adhesiveness between the tendinous stumps.

Suture failure, whether due to stitch rupture or slippage, is one of the concerns in intraoperative tenorrhaphies. In both situations, the tendon may be stretched, with a consequent loss of strength and even complete loss of the repair. One study revealed that slips greater than 3 mm can compromise the repair and are more associated with the need for tenolysis [31]. Another study showed that a spacing greater than 10 mm between the tendon stumps favours re‐rupture in the first 12 months of injury to the calcaneal tendon [39].

Sturdy suture threads produce stronger repairs but can fail due to slipping. Adding stitches and knots, in addition to locking sutures, reduces this type of failure, but causes tendon thickening, potential ischaemic effects and increased tissue reaction [16]. When thickening occurs in tendons that run within sheaths and under pulleys, it may impose restrictions on the structure's movement, eventually causing adhesions and loss of movement [3, 42]. Thus, the tendon repair must involve a suture with great strength, less predisposed to tissue slip, and that produces a minimum reaction in the host. This can enable early rehabilitation onset and the consequent anticipation of functional recovery.

In the present study, a construction using the suture associated with CA was more resistant to rupture, and in several rabbits, the repair failed due to suture rupture (one third of the cases), even without a statistical difference when compared to the group repaired without CA. These data are in line with the findings by Smith et al., who suggested better stitch retention with the use of glue [32].

In the present investigation, the break strength was taken only as a maximum limit, with no failure pattern cyclical effort being performed, due to the lack of such resource. Furthermore, a traction test after 30 days of healing was not done. The findings found are in line with the results obtained in the study by Bala et al, who observed an increase in mechanical resistance in calcaneal tenorrhaphies that used CA [4]. This current study adds a third group in which only CA was used in the repair, which proved insufficient to guarantee mechanical tensile strength.

In the macroscopic pathological study, there was no superficial or deep infection. After euthanasia, 30 days after the first procedure, there were no fistulas, suture dehiscence and superficial or deep necrosis in the operated tendons, with no difference between the groups. The microscopic study comparing the intensity and pattern of inflammatory activity, in addition to aspects related to repair, such as fibrosis, granulomatous reaction and neovascularization, showed a difference between the groups treated with and without CA. These comparison parameters were similar to those reported by Tavares et al. when studying the healing process of rabbit calcaneal tendons [36]. Most tendons with CA used for repair had micro abscesses. Bala et al. had similar findings in the anatomopathological study, observing a process of neovascularization and formation of granulation tissue in the group in which CA was used [4].

The inflammatory activity was more intense in the tendons where the glue was used, with hypercellularity and more disorganized fibres. Within the same stage between the groups, the straight pattern in the axis of movement occurred more frequently in the group with sutures only, which suggests less interference from the glue in this process. The inflammatory process exacerbation can also justify the increase in tendon diameter, which occurred in Groups 2 and 3. These findings are similar to those from the study run by Thermann et al., who found more fibre disorganization (a non‐straight and uniform pattern), more macrophage proliferation and maximum thickening within 4 weeks of the healing process in tendons repaired with the adjuvant use of fibrin glue [37]. The authors also mentioned that, after 12 weeks, the tendons that were conservatively treated, with sutures and with the addition of fibrin glue, already had a similar appearance, both in terms of fibre reorganization and cellularity pattern.

In the present study, tendon thickness was compared only after 30 days of the first procedure, and there was no difference in the diameter of the tendons in the three groups at that time. Evaluation at 12 weeks could show changes in tendon thickness.

Another relevant fact of this study was the existence of necrosis and micro abscess formation in the two groups that used the glue. These changes can be associated with the thermal effect generated by the exothermic polymerization reaction of the adjuvant product. Despite this, none of these groups developed a local or systemic manifestation of infection. The systemic manifestations of infection were evaluated by the animal's behaviour (reclusion and aggressiveness), acceptance of food and reaction to contact with the surgical team. Likewise, it was common to find a granulomatous reaction with epithelioid cells, typical of a response to contain an infectious process.

The resected material was not cultured, which may represent a limitation of the present study, but there were no external phlogistic signs and elements suggestive of infection (necrosis or secretion). One possibility suggested would be that these abscesses and other microscopic changes could be generated by low‐virulence germs, mainly from the animal's skin.

A limitation of this study was that the cyclic stress test was not performed on the tendons, since the final event that promotes tendon rupture is acute eccentric muscle contraction. However, small and multiple eccentric traumas with cyclic manifestations generate substantial damage to the intact tendon and can produce stretching and rupture of the surgical repair. Furthermore, other parameters, such as elasticity and stiffness, were not measured.

It was not the objective of the study to carry out a quantitative analysis of collagen fibres, because it would not add information different from that described in the objectives of the study. However, other more specific techniques, such as immunohistochemical staining or microscopy analysis with second harmonic generation, would be the most recommended. The histochemical evaluation of the operated parts was not addressed. Taking as a concept the alterations present in the repair phase, with elevation of several substances such as platelet‐derived growth factor, bone morphogenetic proteins 12, 13 and 14, fibroblast growth factor and the insulin‐like growth factor (Somatomedin C), these substances were not collected and dosed for comparison between the groups [25]. The glue action can interfere with the formation of these products, changing the time duration and intensity of the healing process.

CAs have been used for decades in medical practice. Initially used by general surgery to repair skin lesions, more recently it has been used in ocular surgery, vascular surgery (vascular sclerosis), maxillofacial, gynaecologist (tubal occlusion) and even in neurosurgery in the treatment of intracranial aneurysms [1, 10, 19, 24, 35]. Its use has proven successful in these areas and seems promising in other applications, expanding its clinical horizon.

In this study, CA 2‐octyl was a low‐cost, safe agent that has not caused local or systemic clinical complications when compared to conventional sutures. Despite increasing the inflammatory process in the repair phase, there was no clinical repercussion when using it. Extrapolating these results to humans, its use can be an option in cases where faster functional recovery is needed, in situations of high demand and, therefore, will be at risk for a second rupture, in addition to those cases where the rate of re‐rupture is higher, such as in diabetic patients.

With the evolution of knowledge on the healing process involving these products, as well as the evolution of CAs and other adjuvants, there may be an improvement in results and a shortening in the recovery process.

## CONCLUSION

2‐octyl CA added immediate mechanical strength to the calcaneal tendon repair in the rabbits, without increasing local complications, despite pathological changes (focal necrosis, micro abscesses and granulomatous reaction) up to 30 days after the repair.

## AUTHOR CONTRIBUTIONS

All authors have read and approved the final manuscript. Conception and design: Rogério de Andrade Gomes, Marco Antônio Percope de Andrade, Luiz Eduardo Moreira Teixeira and Ivana Duval de Araújo. Methodology development: Rogério de Andrade Gomes, Marco Antônio Percope de Andrade, Luiz Eduardo Moreira Teixeira, Bruno Janotti Pádua, Ivana Duval de Araújo and Eduardo Paulino Júnior. Data acquisition: Rogério de Andrade Gomes, Bruno Janotti Pádua, Sidney Max e Silva, Matheus Maciel Vilela, Paulo Feliciano Sarquis Dias and Eduardo Paulino Júnior. Data analysis and interpretation: Rogério de Andrade Gomes, Luiz Eduardo Moreira Teixeira, Bruno Janotti Pádua, Sidney Max e Silva, Matheus Maciel Vilela and Paulo Feliciano Sarquis Dias. Manuscript writing and review: Rogério de Andrade Gomes, Marco Antônio Percope de Andrade, Luiz Eduardo Moreira Teixeira, Bruno Janotti Pádua and Paulo Feliciano Sarquis Dias. Study supervision: Marco Antônio Percope de Andrade, Luiz Eduardo Moreira Teixeira and Ivana Duval de Araújo.

## CONFLICT OF INTEREST STATEMENT

The authors declare no conflicts of interest.

## ETHICS STATEMENT

This study was approved by the Ethics Committee of Animal Experimentation of the Federal University of Minas Gerais (UFMG) (protocol # 390/2017). This study was carried out at the Laboratory of Experimental Surgery of the Medical School of the UFMG.

## Supporting information

Suporting information.

## Data Availability

Data available within Supporting Information.
