# Supplementary material for: 2‐Octyl cyanoacrylate adjuvant use in calcaneal tendon repair: A comparative experimental study in rabbits
Source: J Exp Orthop. 2025 Apr 1;12(2):e70222. doi: 10.1002/jeo2.70222 (PMC11959501; doi:10.1002/jeo2.70222)
Supplement: Supplementary file 1 — Suporting information. [file JEO2-12-e70222-s001.docx]

Suplementary data.

Group 1: Suture

| **RABBIT** | **RUPTURE STRENGTH (N)** | **RUPTURE PADRON** |
| --- | --- | --- |
| **1** | 57.234 | SLIPPAGE |
| **2** | 46.122 | SLIPPAGE |
| **3** | 38.591 | SLIPPAGE |
| **4** | 58.146 | SLIPPAGE |
| **5** | 51.624 | SLIPPAGE |
| **6** | EXCLUDED | EXCLUDED |
| **7** | 60.342 | SLIPPAGE |
| **8** | 49.212 | SLIPPAGE |
| **9** | 45.75 | SLIPPAGE |
| **10** | 55.125 | SLIPPAGE |
| **11** | 37.855 | SLIPPAGE |
| **12** | 48.603 | SLIPPAGE |
| **13** | 68.522 | SLIPPAGE |
| **14** | 111.957 | SLIPPAGE |
| **15** | 57.126 | SLIPPAGE |

Group 2: suture + cyanoacrylate

| **RABBIT** | **RUPTURE STRENGTH (N)** | **RUPTURE PADRON** |
| --- | --- | --- |
| 1 | 47.427 | SLIPPAGE |
| 2 | 46.22 | RUPTURE OF SUTURE |
| 3 | 39.924 | SLIPPAGE |
| 4 | 78.348 | SLIPPAGE |
| 5 | 70.856 | SLIPPAGE |
| 6 | 117.311 | RUPTURE OF SUTURE |
| 7 | 110.368 | SLIPPAGE |
| 8 | 115.301 | RUPTURE OF SUTURE |
| 9 | 50.231 | SLIPPAGE |
| 10 | 108.416 | RUPTURE OF SUTURE |
| 11 | 55.164 | SLIPPAGE |
| 12 | 118.576 | SLIPPAGE |
| 13 | 45.191 | SLIPPAGE |
| 14 | 75.759 | SLIPPAGE |
| 15 | 65.53 | RUPTURE OF SUTURE |

Group 3: cyanoacrylate

| **RABBIT** | **RUPTURE STRENGTH(N)** |
| --- | --- |
| 1 | 35.845 |
| 2 | 22.125 |
| 3 | 18.074 |
| 4 | 12.788 |
| 5 | 18.535 |
| 6 | 12.2 |
| 7 | 12.837 |
| 8 | 25.439 |
| 9 | 11.906 |
| 10 | 21.977 |
| 11 | 18.486 |
| 12 | 8.944 |
| 13 | 9.64 |
| 14 | 7.797 |
| 15 | 9.64 |

Weigh rabbits before the 2nd stage

| Group 1 - Suture | Group 2 - Suture + cyanoacrylate | Group 3 - cyanoacrylate |
| --- | --- | --- |
| 2814 | 2893 | 3190 |
| 2902 | 2660 | 2979 |
| 3064 | 2721 | 3170 |
| 3110 | 3201 | 3019 |
| 2940 | 2928 | 2897 |
| EXCLUDED | 3401 | 2810 |
| 3315 | 3282 | 2907 |
| 2768 | 3350 | 3096 |
| 3180 | 3026 | 2775 |
| 2540 | 3006 | 2898 |
| 2640 | 2701 | 2840 |
| 2615 | 2489 | 2761 |
| 2890 | 3140 | 2776 |
| 2620 | 2283 | 3346 |
| 3300 | 3136 | 3017 |
